# Stabilizing mutation of *CTNNB1*/beta-catenin and protein accumulation analyzed in a large series of parathyroid tumors of Swedish patients

**DOI:** 10.1186/1476-4598-7-53

**Published:** 2008-06-09

**Authors:** Peyman Björklund, Daniel Lindberg, Göran Åkerström, Gunnar Westin

**Affiliations:** 1 Department of Surgical Sciences, Endocrine Unit, Uppsala University, Uppsala University Hospital, SE-751 85 Uppsala, Sweden

## Abstract

**Background:**

Aberrant accumulation of β-catenin plays an important role in a variety of human neoplasms. We recently reported accumulation of β-catenin in parathyroid adenomas from patients with primary hyperparathyroidism (pHPT). In *CTNNB1 *exon 3, we detected a stabilizing mutation (S37A) in 3 out of 20 analyzed adenomas. The aim of the present study was to determine the frequency and zygosity of mutations in *CTNNB1 *exon 3, and β-catenin accumulation in a large series of parathyroid adenomas of Swedish patients.

**Results:**

The mutation S37A (TCT > GCT) was detected by direct DNA sequencing of PCR fragments in 6 out of 104 sporadic parathyroid adenomas (5.8%). Taking our previous study into account, a total of 9 out of 124 (7.3%) adenomas displayed the same mutation. The mutations were homozygous by DNA sequencing, restriction enzyme cleavage, and gene copy number determination using the GeneChip 500 K Mapping Array Set. All tumors analyzed by immunohistochemistry, including those with mutation, displayed aberrant β-catenin accumulation. Western blotting revealed a slightly higher expression level of β-catenin and nonphosphorylated active β-catenin in tumors with mutation compared to those without. Presence of the mutation was not related to distinct clinical characteristics.

**Conclusion:**

Aberrant accumulation of β-catenin is very common in parathyroid tumors, and is caused by stabilizing homozygous mutation in 7.3% of Swedish pHPT patients.

## Background

Parathyroid disease with hypersecretion of parathyroid hormone and generally also hypercalcemia occurs in primary hyperparathyroidism (pHPT), due to growth regulatory disturbance in one or several parathyroid glands. Activation of *CCND1 *oncogene expression or inactivation of the *MEN1 *tumor suppressor gene contributes to deregulated growth control in a fraction of sporadic parathyroid adenomas [1-4].

Activation of the Wnt/β-catenin signaling pathway by aberrant accumulation of stabilized β-catenin is involved in the development of many neoplasms. β-catenin accumulation is typically caused by mutations in components of the signaling pathway, such as APC, Axin, β-Trcp, and WTX, or results from secondary events. In addition, protein stabilizing mutations in the glycogen synthase kinase 3β phosphorylation sites of β-catenin (Ser-33, Ser-37, Thr-41, Ser-45) occur with varying frequency in several neoplasms [5-9].

We recently reported activation of the Wnt/β-catenin signaling pathway by aberrant accumulation of β-catenin in parathyroid adenomas from patients with pHPT [10]. The accumulation of β-catenin was caused by expression of an aberrantly spliced internally truncated Wnt receptor LRP5 or by a stabilizing mutation (S37A) in *CTNNB1 *exon 3 [10,11]. Stabilizing mutations of *CTNNB1 *have not been detected in parathyroid adenomas of patients from Japan and the United States [12,13]. Here we have determined the frequency and zygosity of mutations in exon 3 of *CTNNB1*, and β-catenin expression status in a large series of parathyroid adenomas of Swedish patients.

## Methods

### Tissue Specimens

Sporadic parathyroid adenomas (n = 104) were acquired from 104 Swedish patients with pHPT diagnosed and operated on in the clinical routine at the Uppsala University Hospital. Normal parathyroid tissue was obtained as normal gland biopsies in patients subjected to parathyroidectomy. Tissues were intraoperatively snap frozen. Informed consent and approval of institutional ethical committee were obtained.

### DNA Sequencing

DNA from parathyroid tumors was prepared by standard procedures including proteinase K treatment and phenol extraction. Blood DNA was prepared using the Wizard Genomic DNA Purification Kit (Promega Corp., Madison, WI). DNA was PCR amplified with primers for exon 3 of *CTNNB1*. PCR forward primer: 5'-TGA TGG AGT TGG ACA TGG CC; reverse: 5'-CTC ATA CAG GAC TTG GGA GG. The complementary strand was also sequenced for fragments with mutation. The PCR fragments were sequenced directly on the 3130*xl *Genetic Analyzer using the ABI Prism Dye Terminator Cycle Sequencing Ready Reaction kit (Applied Biosystems, Foster City, CA).

### Restriction Enzyme Digestion

*CTNNB1 *exon 3 PCR fragments were purified using the GFX PCR DNA and Gel Band Purification Kit (GE Healthcare Europe GmbH, Uppsala, Sweden) and cleaved with Xma I or Nla III according to instructions by the manufacturer (New England Biolabs, Inc., Beverly, MA). Products were analyzed by agarose gel electrophoresis.

### CTNNB1 Gene Copy Number

Tumor (S37A) and blood DNA from 4 patients were extracted as described above. DNA was marked with fluorescence dye and hybridized to the Affymetrix GeneChip Mapping 500 K Set Arrays 250K_Nsp_SNP and 250K_Sty_SNP according to the manufacturer's instructions, and analysed by GeneChip Genotyping Analysis Software (GTYPE) using Chromosome Copy Number Analysis Tool (CNAT) (Affymetrix, Inc. Santa Clara, California, USA). Informative SNPs used in the gene copy number determination are shown in Table 1. The experiment was performed at the Bioinformatics and Expression Analysis core facility at NOVUM, Karolinska Institute, Huddinge, Sweden.

**Table 1:** SNP genotyping

**SNP position**	**refSNP ID**	**Patient 1**	**Patient 2**	**Patient 3**	**Patient 4**
41205658	rs7630377	ECN	ECN	ECN	ECN
41209520	rs9859392	ECN	ECN	ECN	ECN
41218746	rs3915129	ECN	NI	ECN	ECN
41237448	rs13072632	ECN	ECN	ECN	ECN
41243358	rs11564447	ECN	ECN	NI	ECN
41262697	rs9824212	ECN	ECN	ECN	ECN
41268711	rs1880480	ECN	ECN	ECN	ECN

Constitutional DNA and parathyroid adenoma DNA with *CTNNB1 *mutation S37A from 4 pHPT patients were genotyped with the GeneChip 500 K Mapping Array Set. The *CTNNB1 *gene is located between positions 41216016 and 41256928 . ECN, equal copy number. NI, non-informative.

### Immunohistochemistry and Western Blotting

Frozen tissue sections were stained as described [10] using an anti-β-catenin goat polyclonal antibody with an epitope mapping at the C-terminus (Santa Cruz Biotechnology, INC., Santa Cruz, CA; catalog no. sc-1496). Protein extracts for Western blotting were prepared [10] in Cytobuster Protein Extract Reagent (Novagen Inc., Madison, Wisconsin, USA) supplemented with Complete protease inhibitor cocktail (Roche Diagnostics GmbH, Penzberg, Germany). The anti-active (nonphosphorylated) β-catenin [14] mouse monoclonal antibody (Upstate, Lake Placid, USA, # 05-665), the anti-β-catenin goat polyclonal antibody (above), and anti-actin goat polyclonal antibody (Santa Cruz Biotechnology INC.) were used. After incubation with the appropriate secondary antibodies, bands were visualized using the enhanced chemiluminescence system (GE Healthcare Europe GmbH, Uppsala, Sweden). Membranes were scanned by the ChemiDoc XRS and the band intensities were determined using Quantity One Software (Bio-Rad Laboratories, Inc., Hercules, California, USA).

### Statistical Analyses

Unpaired *t *test, *z *test, and χ^2 ^test were used. The data were calculated with Statistica 6 (StatSoft, Tulsa, OK, USA). Values are presented as arithmetrical mean ± SEM.

## Results

### Homozygous CTNNB1 Stabilizing Mutation S37A

**Figure 1:**
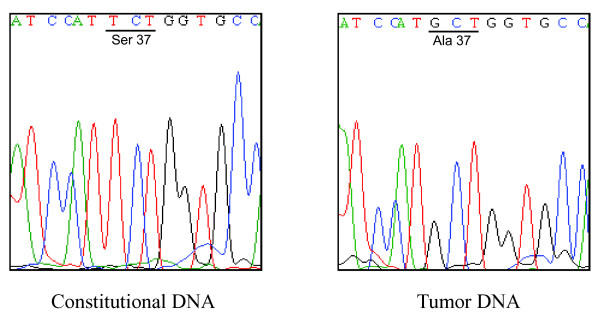
**Representative results of direct DNA sequencing of *CTNNB1 *exon 3**. Constitutional DNA from blood (left panel) and parathyroid adenoma (right panel) of the same pHPT patient.

DNA sequencing analysis detected the *CTNNB1 *stabilizing mutation S37A (TCT > GCT) in 6 out of the 104 (5.8%) analyzed parathyroid adenomas (Figure 1). Constitutional DNA (blood) from 4 out of the 6 patients were available, and encoded the wild-type *CTNNB1 *sequence. The mutations were apparently homozygous by DNA sequencing (Figure 1), as we reported previously for 3 out of 20 adenomas [10]. Taking our previous study into account [10], a total of 9 out of 124 (7.3%) randomly selected parathyroid adenomas displayed the *CTNNB1 *stabilizing mutation S37A.

**Figure 2:**
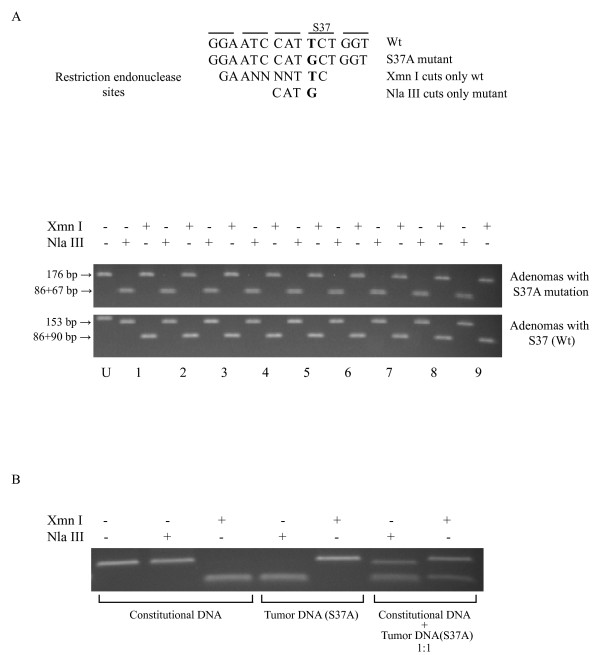
**Analytical restriction enzyme cleavage analysis**. (A), *CTNNB1 *exon 3 PCR fragments (176 bp) were digested with Xmn I or Nla III that cuts only wild-type or only S37A mutant sequences, respectively. Three out of the nine parathyroid adenomas with S37A mutation were identified in our previous study [10]. Nla III cuts also outside of codon 37, close to the fragment end (23 bp). U; uncleaved *CTNNB1 *exon 3 PCR fragment. (B), *CTNNB1 *exon 3 was PCR amplified from a 1:1 mixture of constitutional DNA and tumor DNA with the S37A mutation. The fragment was analyzed by restriction enzyme digestions as in (A).

Homozygosity for the mutation was further substantiated by analytical restriction enzyme digestions. As expected, all 9 fragments harbouring S37A were completely cleaved by Nla III and not by Xma I (Figure 2A). Vice versa was observed for fragments with the wild-type codon S37. PCR amplified fragments were used for the DNA sequencing and restriction analyses, and PCR reactions could in theory favour the mutant allele(s). Unbiased PCR reactions were however confirmed by performing PCR amplification in a 1:1 mixture of constitutional DNA and S37A mutant tumor DNA, and by subsequent analytical restriction enzyme digestion (Figure 2B).

In order to resolve the issue of zygosity for the S37A mutation, 4 tumor DNAs with the corresponding constitutional DNAs were genotyped with the GeneChip 500 K Mapping Array Set (Affymetrix). Gene copy number analysis was done by comparison of informative single-nucleotide polymorphisms (SNPs). Five informative SNPs in *CTNNB1 *and 2 SNPs downstream of the gene showed equal copy number for the 4 paired DNA samples (Table 1). Thus, taking also the DNA sequencing results into account the S37A mutation was homozygous in these 4 tumours, rather than hemizygous with one mutant and one deleted *CTNNB1 *allele.

### β-catenin Protein Expression

**Figure 3:**
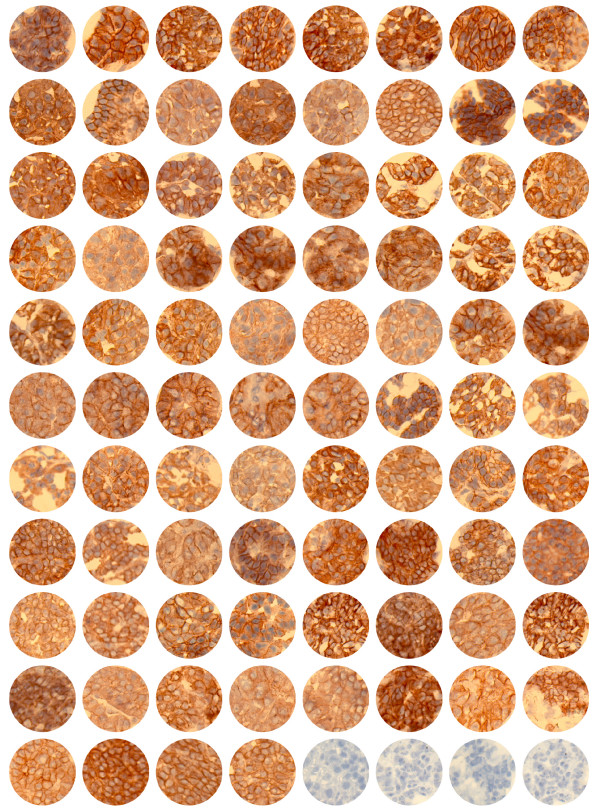
**Immunohistochemical analysis of β-catenin**. Immunostaining of 84 parathyroid adenomas with an anti-β-catenin goat polyclonal antibody. The first 9 tumor specimens, of which 3 were described previously [10], displayed *CTNNB1 *homozygous stabilizing mutation S37A. No staining was seen in the absence of primary antibodies (last 4 specimens).

**Figure 4:**
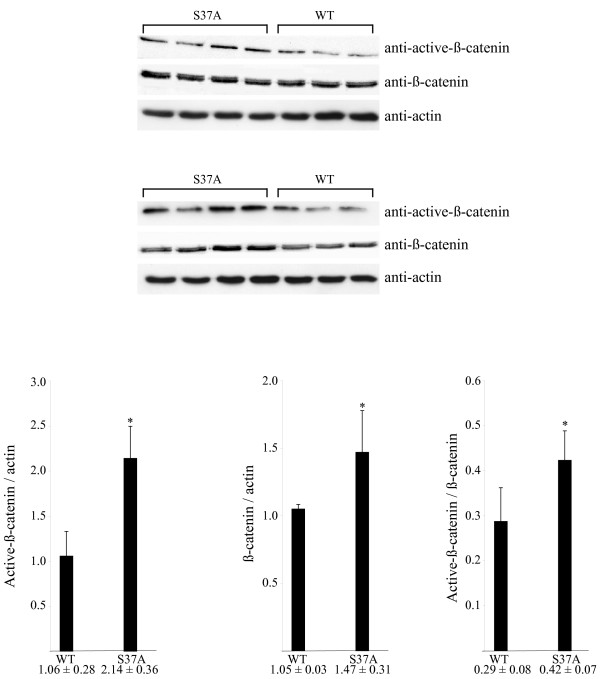
**Western blotting analysis of total and non-phosphorylated active β-catenin**. Eight pHPT tumors with *CTNNB1 *mutation S37A and six pHPT tumors without mutation were analyzed. The group of six tumors expressed the internally truncated LRP5 receptor [11]. All analyzed tumors showed accumulation of active β-catenin in comparison to normal parathyroid tissue (not shown). An anti-active (nonphosphorylated) β-catenin monoclonal antibody [14] and a goat polyclonal antibody with an epitope mapping at the C-terminus of β-catenin were used. Quantisation of the obtained signals are shown below. *, p < 0.05.

Previously, we reported aberrant accumulation of β-catenin in all (n = 37) analyzed parathyroid adenomas [10]. Of the tumors analyzed here by DNA sequencing, 81 frozen parathyroid adenomas, including 6 with the S37A mutation, were of sufficient good quality for immunohistochemical analysis with a β-catenin goat polyclonal peptide antiserum [10]. The three pHPT tumors with S37A mutation described previously [10] were also included. In addition to membraneous staining, all 84 tumors displayed distinct cytoplasmic/nuclear immunoreactivity (Figure 3). Western blotting analysis was performed to compare the expression level of β-catenin as well as of nonphosphorylated active β-catenin (ratio of β-catenin to actin) in tumors with (n = 8) and without (n = 6) the stabilizing mutation S37A (Figure 4). The six tumors without β-catenin stabilizing mutation all expressed the internally truncated LRP5 receptor [11], and as expected [10,11] all fourteen tumors showed accumulation of nonphosphorylated active β-catenin in comparison to normal parathyroid tissue (not shown). A small but significantly higher expression level was observed of both β-catenin and nonphosphorylated active β-catenin in tumors with stabilizing mutation S37A in comparison to those with wild type β-catenin. The ratio of nonphosphorylated active β-catenin to β-catenin was also slightly higher in tumors with β-catenin stabilizing mutation (Figure 4). No particular clinical characteristics, including age, sex, serum calcium level, serum PTH level or gland weight related to presence of the S37A mutation.

## Discussion

We have found that a total of 9 out of 124 (7.3%) randomly selected parathyroid adenomas displayed the *CTNNB1 *stabilizing homozygous mutation S37A. None of these 9 tumors expressed the internally truncated LRP5 receptor [11] (data not shown), further emphasizing the previous observation that these events are mutually exclusive [11]. The mutated LRP5 receptor, with the central region deleted, is expressed in the majority of pHPT tumors and is required for accumulation of β-catenin and parathyroid tumor cell growth [11].

The S37A *CTNNB1 *mutation commonly occurs also in gastrointestinal carcinoid tumors where 26 out of 29 tumors with mutations harboured S37A [15]. The mutant protein shows resistance to ubiquination and proteosomal degradation, with a longer half-life than wild-type β-catenin. S37A β-catenin also shows an enhanced affinity for LEF1 and TCF4, its DNA-binding partners in transcriptional regulation [16-18]. Homozygous mutation as detected by direct DNA sequencing of *CTNNB1 *seems to be uncommon in other neoplasms, but have been described in a rectal carcinoid tumor and in colorectal cancer [15,19]. To be conclusive regarding zygosity, direct DNA sequencing clearly requires low or no contamination of normal cell populations in the tumor sample. In colorectal cancer cells with inactivating *APC *hemizygous mutation or activating *CTNNB1 *heterozygous mutation, the total β-catenin signaling activity seemed dependent also on silencing of *SFRP *genes by promoter hypermethylation with consistent constitutive WNT signaling [20]. Whether combined activity of two mutant S37A *CTNNB1 *alleles suffices for benign parathyroid tumor growth or whether constitutive WNT signaling is required in addition, remain to be investigated.

DNA sequence analysis of 24 parathyroid adenomas from Japanese patients revealed no *CTNNB1 *mutations, and immunohistochemistry showed weak cytoplasmic β-catenin staining in 2 tumors [12]. In another study from Japan (n = 9), cytoplasmic and/or membranous β-catenin staining was seen in 8 adenomas and nuclear staining in one adenoma. DNA sequencing analysis was not done in these specimens [21]. Furthermore, a recent study did not detect *CTNNB1 *exon 3 mutations in 97 sporadic parathyroid adenomas from patients who had undergone parathyroidectomy in the United States. Unfortunately, β-catenin protein expression was not evaluated in this report [13]. Since we observed a *CTNNB1 *mutation frequency of 7.3% in adenomas from Swedish patients, this may suggest possible contribution of geographical origin (dietary or environmental differences, or different genetic backgrounds) to mechanisms of parathyroid disease. The *CTNNB1 *mutation frequency vary considerably also in colorectal cancer (1–60%) and melanomas (0.02–27%), apparently not related to geographical origin [8,17,22-30]. The observations may be attributed to the stochastic distribution of probability in analyzed material or to other causalities, like that the tumor sample purity and pathology must be guaranteed.

## Conclusion

By analyzing a large series of tumors from Swedish patients, this study further emphasizes β-catenin accumulation as the most common aberration in parathyroid tumors of primary origin. *CTNNB1*-stabilizing mutations were found in 5.8% of the tumors, or in 7.3% when taking our previous study [10] into account. The WNT/β-catenin signaling pathway, with β-catenin and the internally truncated LRP5 receptor [11] in particular, present therapeutic targets for hyperparathyroidism.

## Competing interests

The authors declare that they have no competing interests.

## Authors' contributions

PB participated in the design of the study, performed experiments, analyzed data and performed the statistical analysis, DL analyzed the data, GÅ helped to draft the manuscript, GW conceived of the study, participated in its design and coordination and drafted the manuscript. All authors read and approved the final manuscript.
